# Effect of Substrate Stiffness on Physicochemical Properties of Normal and Fibrotic Lung Fibroblasts

**DOI:** 10.3390/ma13204495

**Published:** 2020-10-10

**Authors:** Joanna Raczkowska, Barbara Orzechowska, Sabina Patryas, Kamil Awsiuk, Andrzej Kubiak, Masaya Kinoshita, Masami Okamoto, Justyna Bobrowska, Tomasz Stachura, Jerzy Soja, Krzysztof Sładek, Małgorzata Lekka

**Affiliations:** 1 The Marian Smoluchowski Institute of Physics, Jagiellonian University, Łojasiewicza 11, 30-428 Kraków, Poland; sabina.patryas@student.uj.edu.pl (S.P.); kamil.awsiuk@uj.edu.pl (K.A.); 2 Institute of Nuclear Physics Polish Academy of Sciences, Radzikowskiego 152, 31-342 Kraków, Poland; barbara.orzechowska@ifj.edu.pl (B.O.); andrzej.kubiak@ifj.edu.pl (A.K.); justyma.bobrowska@ifj.edu.pl (J.B.); malgorzata.lekka@ifj.edu.pl (M.L.); 3 Toyota Technological Institute, 2-12-1 Hisakata, Tempaku, Nagoya, 468-8511, Japan; sd18408@toyota-ti.ac.jp (M.K.); okamoto@toyota-ti.ac.jp (M.O.); 4 2nd Department of Internal Medicine, Jagiellonian University Medical College, Jakubowskiego 2, 30-688 Kraków, Poland; tomasz.stachura@interia.pl (T.S.); jerzysoja@op.pl (J.S.); mmsladek@cyf-kr.edu.pl (K.S.)

**Keywords:** idiopathic pulmonary fibrosis, fibroblasts, substrate elasticity, force spectroscopy

## Abstract

The presented research aims to verify whether physicochemical properties of lung fibroblasts, modified by substrate stiffness, can be used to discriminate between normal and fibrotic cells from idiopathic pulmonary fibrosis (IPF). The impact of polydimethylsiloxane (PDMS) substrate stiffness on the physicochemical properties of normal (LL24) and IPF-derived lung fibroblasts (LL97A) was examined in detail. The growth and elasticity of cells were assessed using fluorescence microscopy and atomic force microscopy working in force spectroscopy mode, respectively. The number of fibroblasts, as well as their shape and the arrangement, strongly depends on the mechanical properties of the substrate. Moreover, normal fibroblasts remain more rigid as compared to their fibrotic counterparts, which may indicate the impairments of IPF-derived fibroblasts induced by the fibrosis process. The chemical properties of normal and IPF-derived lung fibroblasts inspected using time-of-flight secondary ion mass spectrometry, and analyzed complexly with principal component analysis (PCA), show a significant difference in the distribution of cholesterol and phospholipids. Based on the observed distinctions between healthy and fibrotic cells, the mechanical properties of cells may serve as prospective diagnostic biomarkers enabling fast and reliable identification of idiopathic pulmonary fibrosis (IPF).

## 1. Introduction

Interstitial lung diseases (ILD) form a heterogeneous group of disorders with varying degrees of inflammation and fibrosis, affecting mainly the interstitium of the lungs (the tissue and space between the epithelial and endothelial basement membranes) [1,2]. Though ILD may be caused by some specific factors such as connective tissue diseases, diseases with granuloma formation, organic dust, or certain drugs, in the majority of cases, no obvious cause can be identified [3]. One of the most frequent forms of ILDs is idiopathic pulmonary fibrosis (IPF) [4,5,6,7].

Nowadays, the diagnostic process is based on a multidisciplinary approach involving pulmonologist, radiologist and pathologist experts and sophisticated examination techniques, such as high-resolution computed tomography (HRCT), and in some cases lung biopsy [7,8,9,10], which makes it long, expensive and, unfortunately, still not always reliable.

Making the proper diagnosis of IPF is hard. There are almost 200 different ILDs, and in most cases, their clinical manifestations are very similar if not the same. Additionally, there are a lot of similarities and overlap of the radiologic and histopathological patterns, which makes diagnosis even more difficult. In the year 2018, a multidisciplinary committee of IPF experts from the American Thoracic Society, European Respiratory Society, Japanese Respiratory Society, and Latin American Thoracic Society provided ‘An Official ATS/ERS/JRS/ALAT Clinical Practice Guideline’ formulating recommendations related to the diagnosis of IPF [11]. However, even with the proposed interdisciplinary approach, significant disagreement in diagnosed disease exists within physicians, especially between community- and academic-based ones; therefore, the diagnosis of an individual patient can significantly differ depending on the physician and, particularly, the location of the evaluation. Moreover, in clinical practice, multidisciplinary experts rarely work together and usually the pulmonary physician is responsible for integration and analysis of all the gathered clinical, radiographic, and pathologic data and final diagnosis of a given patient. Therefore, there is a constant need for an earlier, non-invasive and reliable diagnostic methods [12], crucial for effective therapies. A fast diagnosis enables the early treatment of IPF, which is critical to preserving the patients’ lung function, reduce the risk of acute exacerbations, and improve outcomes [12,13].

In IPF, the changes in cell properties, together with an extensive deposition of collagen and other components of the extracellular matrix (ECM), disrupt normal lung architecture and functions. The progression of fibrosis and inflammatory processes may lead to the development of dyspnea, cough, and ultimately, in some cases, respiratory failure and death [14]. Although the etiology of IPF remains poorly defined, it is believed to be the result of repeated damage to the alveolar epithelium and aberrant wound healing processes in the pulmonary interstitium [15]. The progress of IPF might be associated with an aberrant wound healing process in the pulmonary interstitium, due to fibroblast proliferation and the abnormal accumulation of ECM [16,17,18]. Moreover, the increased matrix stiffening observed in the lung fibrotic process may be a critical fibrogenesis driving factor [19] and the myofibroblast differentiation can be induced in fibroblasts, merely by altering the stiffness of the underlying substrate [20]. In turn, the stiffening of single fibroblasts may affect the lungs at a tissue level, leading to the corruption of mechanically-derived signals that are typically transmitted through the ECM [21,22]. Normal pulmonary function and architecture of lungs may be influenced by the rapid, slow, or mixed [2,23] progress of the disease, leading to an irreversible increase of stiffness, even up to 30-fold [24,25,26]. A panel of IPF researchers gathered in 2014 by the National Heart, Lung, and Blood Institute in the United States emphasized that the mechanisms of matrix stiffening and influence of increased stiffness on cellular processes remain an important, open question in the field of IPF research [27].

A lot of effort is made in the development of the prognostic indicators of IPF [2,23]. The cytoskeleton organization, as well as the contractility of the lung fibroblast originating from IPF, has been reported to differ from the normal tissue [28,29]. This implies that the mechanical properties of individual fibroblasts should be altered. Besides, the mechanical properties of the fibroblasts’ environment seem to be an extremely important factor in cellular response, thus, this requires a deeper study of the effect of substrate stiffness on properties of lung fibroblasts towards establishing its role in the development of IPF lung impairment. However, up to date, the research aimed at this issue focuses mainly on the elasticities typical for healthy and fibrotic lung fibroblasts, i.e., up to a few tens of kPa [21,25,29,30,31]. They show the great impact of stiffness on cellular behavior, but the underlying mechanisms are still not known. In turn, some other research report that besides substrate stiffness, several other material properties including the damping coefficient [32] and stress relaxation [33] of the substrates are of importance, and should be considered in the mechanotransduction mechanism [34].

In our previous work [35], we have shown that the elasticity of polydimethylsiloxane (PDMS) substrates has an impact on lung fibroblasts response affecting their properties and proliferation. Thus, we assume that these differences may be potentially useful to discriminate between healthy and fibrotic fibroblasts. To verify this hypothesis, we decided to expand the study and perform detailed tests of the impact of substrate stiffness on physicochemical properties of well-defined, commercially available, immortalized cell lines of normal and IPF-derived lung fibroblasts. In the present paper, the proliferation and elastic modulus of normal and IPF lung fibroblasts were studied using fluorescence microscopy (MF) and atomic force microscopy (AFM) working in a force spectroscopy mode, respectively. The proliferation of alive cells was examined using MTS assay. To compare the chemical properties of normal and IPF-derived lung fibroblasts, their chemical composition was inspected using time-of-flight secondary ion mass spectrometry (ToF-SIMS), combined with principal component analysis (PCA), which allows one to detect subtle differences between the samples.

## 2. Materials and Methods

### 2.1. Preparation of PDMS Substrates

Sylgard 184 (Dow Corning) was used to prepare PDMS substrates. First, the elastomer base was mixed with the curing agent (mass ratio of 10:1). Then, to enable the adjusting of substrate elasticity, a solution of benzophenone (Sigma-Aldrich, Darmstadt, Germany) in xylene (200 mg/mL, POCH Gliwice, Poland) was added to the mixture, at the mass ratio of 1:100 benzophenone:PDMS and degassed. The PDMS substrates were fabricated using a spin-coating technique (spinning speed 500 rpm, KW-4A, Chemat Scientific, Northridge, CA, USA) on a 25 mm round coverslip glass. To produce soft substrates, some samples were additionally irradiated for 5 h with UV light (λ = 254 nm 400 W mercury lamp). In the last step, substrates were baked for 15 min at 150 °C (MCS67 hot plate, CAT, Ballrechten-Dottingen, Germany).

### 2.2. Cell Culture

Human lung fibroblasts (normal LL24, catalog number ATCC-CCL-151 and IPF-derived LL97A, catalog number ATCC-CCL-191 cells) were purchased from ATCC (Manassas, VA, USA). Cells were cultured in the F-12K medium (ATCC, catalog number 30-2004), which was supplemented with a 15% fetal bovine serum (Sigma-Aldrich, Darmstadt, Germany, catalog number: F9665) and 1% antibiotic (Sigma-Aldrich, Darmstadt, Germany, catalog number: P4083) in culture flasks, in a CO_2_ incubator providing 95% air/5% CO_2_ atmosphere. The PDMS substrates which were attached to glass coverslips were placed into the bottom of the Petri dish (35 mm in diameter) and they were sterilized for one hour under UVC light (germicidal lamp, λ = 254 nm) under a laminar flow chamber (Nu425, NuAire, Plymouth, MN, USA). After that, cells (80,000 cells per ml of the culture medium) were placed over the all type of PDMS surface. Next, the Petri dishes were incubated in the CO_2_ incubator by 1, 3, or 6 days. For each experimental sequence, two or three identical samples were prepared and measured. All experiments were repeated at least three times for each cell line and a time-point to proving the reproducibility of the results.

### 2.3. Force Spectroscopy

Commercially available AFM system (Bruker-JPK, Berlin, Germany) equipped with two heads CellHesion or Nanowizard 4 was used to conduct force spectroscopy and quantitative imaging (QI), respectively. The PDMS substrates with cells were mounted in the AFM liquid cell filled with a culture medium (F-12K medium). The commercially available silicon nitride AFM probe (ORC8-10 D, Bruker) was immersed into medium and brought close to the surface. Force curves (i.e., dependencies between a vertical deflection and scanner position) were collected within a grid of 4 × 4 points, forming a 5 µm × 5 µm elasticity map. Each map was recorded for only one cell over the cellular nuclear region. In total, at least 40 cells were measured for each substrate and time point. To obtain the value of the relative Young’s modulus, an approach part of a recorded force curve was analyzed. It was converted into a force versus indentation curve to which the Hertz contact model was fitted [36]. The shape of the probing tip was approximated by a paraboloid. The Young modulus was calculated as a mean ± standard deviation. To validate the statistical significance among various samples, the one-way ANOVA followed by Bonferroni’s post hoc comparisons tests were used.

Local elasticity maps of normal and IPF-derived fibroblasts were recorded using the quantitative imaging (QI) capability of the Nanowizard 4 AFM head. These maps were recorded within a scan area of 50 µm × 50 µm (256 × 256 pixels).

### 2.4. Time of Fight Secondary Ion Mass Spectrometry (ToF-SIMS)

Imaging and molecular characterization of normal and IPF-derived fibroblasts were performed using TOF-SIMS 5 apparatus (ION-TOF GmbH, Munster, Germany). Bi_3_ ion clusters generated by 30 keV bismuth liquid metal ion gun were used as primary ions. The ion dose density was kept below 10^12^ ion/cm^2^ to ensure static mode conditions and a pulsed low-energy electron flood gun was used for charge compensation.

Firstly, high resolution mass spectra (m/Δm > 7000) were acquired from at least 4 randomly chosen, non-overlapping spots (150 μm × 150 μm area). Next, collected mass spectra were analyzed using principal component analysis (PCA), to find alterations in surface chemical properties among the studied LL24 and LL97A cell lines. PCA was conducted using the PLS Toolbox (Eigenvector Research, Manson, WA, USA) for MATLAB (MathWorks, Inc., Natick, MA, USA). Prior to PCA, the intensities of selected peaks from each spectrum were normalized to the sum of selected peaks and mean-centered. Finally, high-resolution images of both cell lines were collected in imagine mode from several non-overlapping 300 μm × 300 μm areas (with a resolution of 256 × 256 points).

### 2.5. Colorimetric MTS Assay

The viability of cells was verified using an MTS calorimetric test. Briefly, fibroblasts were cultured in a multi-well plate (24 wells) in 1 mL of the corresponding culture medium. Next, 100 μL of MTS reagent (tetrazolium compound) was added to the cells in the culture medium. Then, cells were incubated at 37 °C in 95% air/5% CO_2_ atmosphere, in the incubator (Nuaire) for 1 h. The MTS method is based on the reduction of tetrazolium compound by viable cells to generate a colored formazan product that is soluble in cell culture media. The final volume of 1.1 mL was pipetted to a 96-well plate with 100 μL per hole. The absorbance was determined in the 96-well for each time spot of 24 h, 72 h, and 144 h at OD = 490 nm. The MTS assay was repeated at least three times for each cell line and a time-point. For each experimental run, two or three identical samples were prepared and measured.

### 2.6. Fluorescence Imaging

Before fluorescence staining of actin filaments and the cell nucleus, cultured cells were pre-fixed to the substrate by adding a 1 mL solution of 3:7% of paraformaldehyde (Fluka, Charlotte, NC, USA) to the culture medium for 2 min at 37 °C. Then, cells were washed with phosphate-buffered saline (PBS, Sigma-Aldrich, Darmstadt, Germany) 3 times for 2 min. Afterwards, the sample was immersed in the solution of 3.7% of paraformaldehyde Fluka, Charlotte, NC, USA) for 15 min at room temperature, to fix cells firmly. After fixation, coverslips with cells were rinsed twice with the PBS buffer for 2 min. After that, a cold solution (4 °C) of 0.2% Triton X-100 (Sigma-Aldrich, Darmstadt, Germany) was added for 4 min, followed by washing coverslips with the PBS buffer for 2 min. Next, cells were incubated with phalloidin conjugated with a 1:200 solution Alexa Fluor 488 dye (Invitrogen, Carlsbad, CA, USA) for 40 min, and subsequently, cell nuclei were incubated with a 1:5000 solution containing Hoechst dye (Sigma) for 14 min. The fluorescent images were collected from at least three repetitions carried out for each cell line and a time-point. For each experimental run, at least 10 fluorescent images from two or three coverslips with stained cells were collected.

## 3. Results

### 3.1. Fluorescence

**Figure 1:**
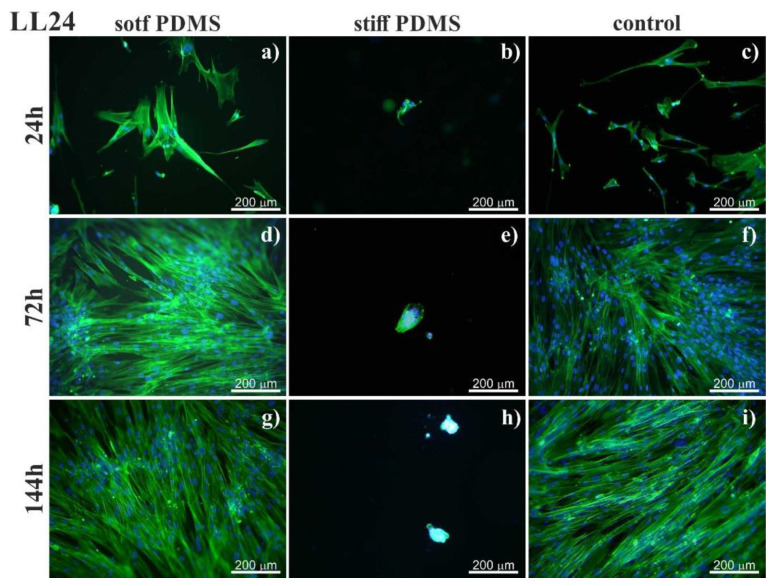
Normal lung fibroblasts (LL24) visualized by fluorescent microscopy by staining actin cytoskeleton (phalloidin—Alexa Fluor 488) and cell nucleus (Hoechst). Cells cultured on soft (left column) and stiff (central column) polydimethylsiloxane (PDMS) were compared to cultures carried out on the glass (control; right column). Cultures were conducted for 24h (**a–c**), 72h (**d–f**) and 144h (**g–i**).

**Figure 2:**
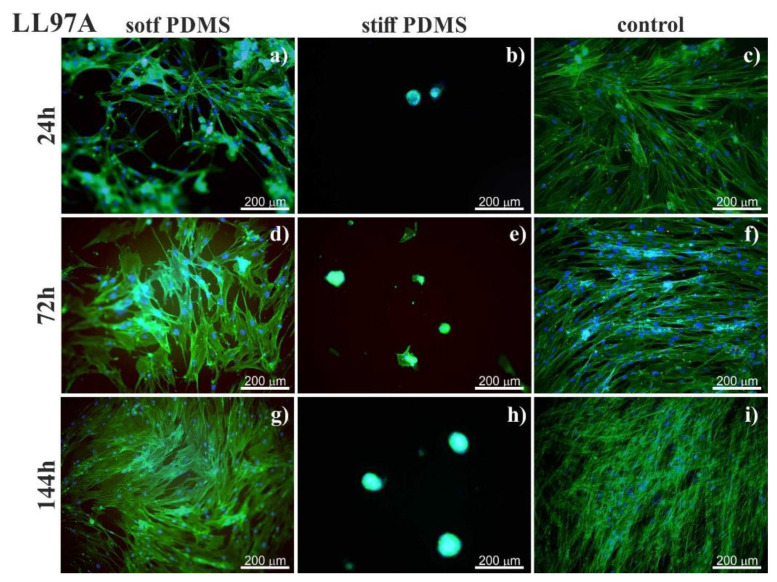
Fluorescent images of idiopathic pulmonary fibrosis (IPF)-derived lung fibroblasts (LL97) visualized by staining actin cytoskeleton (phalloidin—Alexa Fluor 488) and cell nucleus (Hoechst). Cells cultured on soft (left column) and stiff (central column) PDMS were compared to cultures carried out on the glass (control; right column). Cell culture was conducted (right column) after incubation for 24 (**a–c**), 72 (**d–f**) and 144h (**g–i**).

To elaborate on how fibroblasts behave in a distinct mechanical environment, two PDMS substrates characterized by 600 kPa (referred to here as a soft PDMS substrate) and 1.5 MPa (a stiff PDMS) were chosen. The spreading of fibroblasts was monitored by fluorescence imaging after 24h, 72 h and 144 h of culture (Figure 1 and Figure 2).

**Figure 3:**
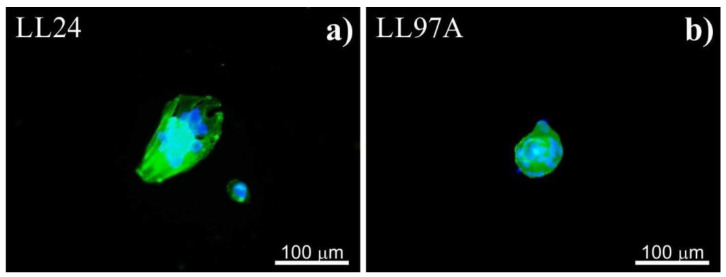
Agglomerates of healthy (LL24, **a**) and fibrotic (LL97A, **b**) lung fibroblasts after 72 h culture on stiff PDMS substrate.

For stiff PDMS substrate (of 1.5 MPa), instead of spreading, cells tend to aggregate and to form agglomerates, with cells growing on each other, as shown in Figure 3.

### 3.2. MTS

**Figure 4:**
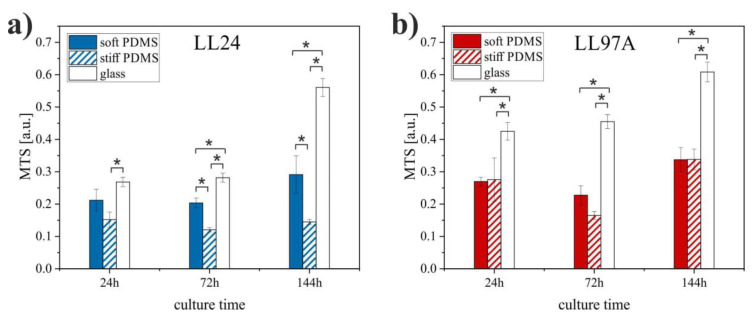
Viability of LL24 (**a**) and LL97A (**b**) cells cultured on soft and stiff PDMS substrates as well as control glass sample after fibroblasts culture for 24, 72, and 144 h obtained using MTS assay (*significantly different from each other, *p* < 0.01). Data are presented as a mean ± standard deviation (SD) from n = 6 repetitions.

In our next step, we elaborate on how the elasticity of the PDMS substrates affects the proliferation of alive fibroblasts, corresponding to the viability of cells using an MTS assay applied at each time spot. The results are presented in Figure 4 for LL24 (a) and LL97A (b) lung fibroblasts.

The MTS values show that the proliferation of healthy lung fibroblasts (Figure 4a) is significantly reduced on the stiffer substrate as compared to the soft one. In contrast, for IPF-derived fibroblasts (Figure 4b), no significant difference is revealed for cells cultured on stiff and soft PDMS. Both cell lines proliferate most effectively on control glass samples.

### 3.3. Elasticity

**Figure 5:**
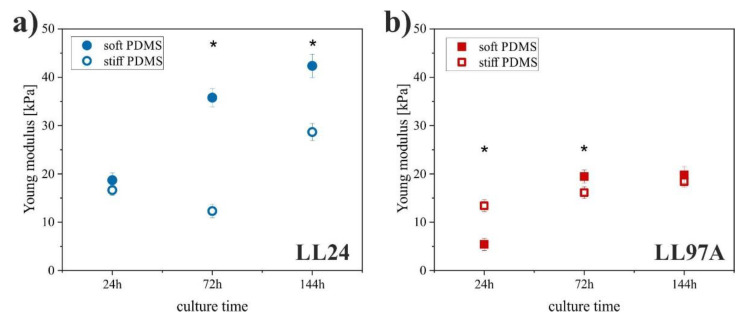
Young modulus of LL24 (**a**) and LL97A (**b**) cells on soft (solid symbols) and stiff (open symbols) PDMS after incubation for 24, 72, and 144h (*significantly different from each other, *p* < 0.01) determined using atomic force microscopy (AFM)-based force spectroscopy. Error bars indicate the SD (n ≥ 40).

The Young modulus of lung fibroblasts (Figure 5) was determined using the AFM-based force spectroscopy [37] from measurements conducted for all culture times and both PDMS substrates. In this technique, the analysis of the collected force curves with Hertz contact mechanics provides Young modulus as a characteristic property for a material with given mechanical properties.

The LL24 fibroblasts cultured on soft PDMS showed a significantly increased Young modulus after 72 and 144 h as compared to the stiff PDMS. These results differ noticeably from the observations made for the LL97A fibroblasts. Here, a significantly increased Young modulus on the stiff PDMS is observed at early incubation times (24 h), and for longer culture times (72 h, 144 h), the stiffness of LL97A fibroblasts becomes comparable for both analyzed substrates.

### 3.4. Elasticity Maps

**Figure 6:**
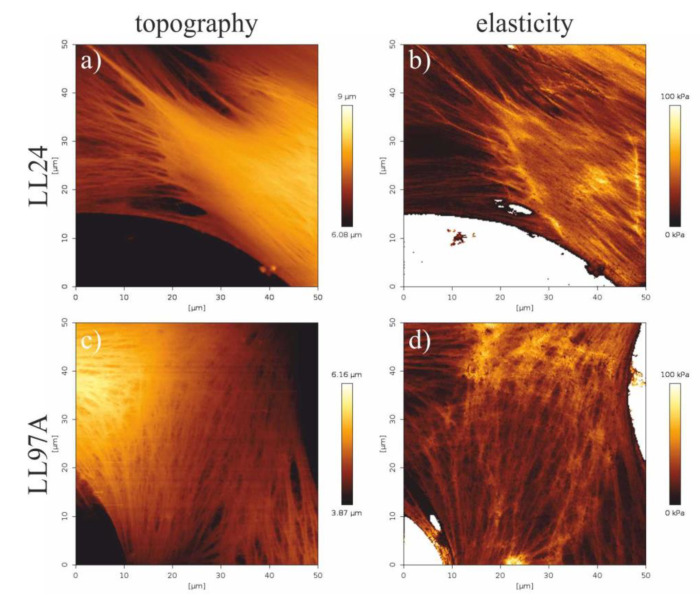
Topography (**a**,**c**) and elasticity maps of fibroblasts fragments (**b**,**d**) recorded for healthy (LL24, **a**,**b**) and IPF-derived (LL97A, **c**,**d**) fibroblasts recorded using the quantitative imaging (QI) mode of AFM.

In addition to the mean elasticity, the spatial distribution of mechanical properties of cells was also analyzed, by recording the elasticity maps of cell fragments (Figure 6).

A comparison of the elasticity maps recorded for a healthy LL24 cell line (Figure 6b) and IPF-derived LL97A one (Figure 6d) shows slight differences in the spatial distribution of their mechanical properties. For healthy fibroblasts, elasticity is highly heterogeneous, rather small for the central part of the cell with limited stiffer regions, located mostly at the edges of a cell. In turn, maps recorded for IPF-derived fibroblasts show a highly uniform distribution of elastic properties, with stiffer areas arranged in characteristic fibers in the whole area of the cell.

### 3.5. ToF-SIMS

The possibility of discrimination between normal and fibrotic fibroblasts based on their chemical composition was verified using ToF-SIMS. Prior to the measurements, a drying protocol using multistep washing of cells in diluted solutions of anhydrous alcohol was applied [38].

**Figure 7:**
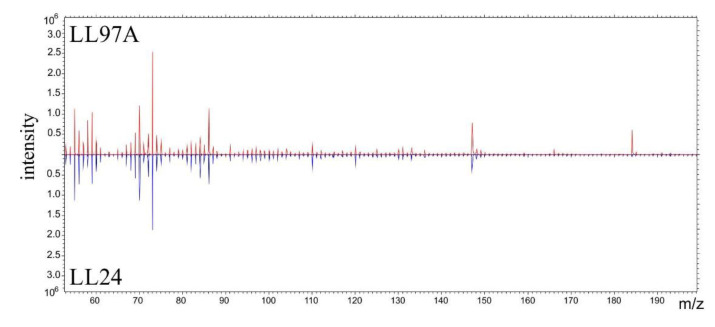
Characteristic spectra recorded for LL24 (**blue line**) and LL97A (**red line**) cells using time-of-flight secondary ion mass spectrometry (ToF-SIMS).

**Figure 8:**
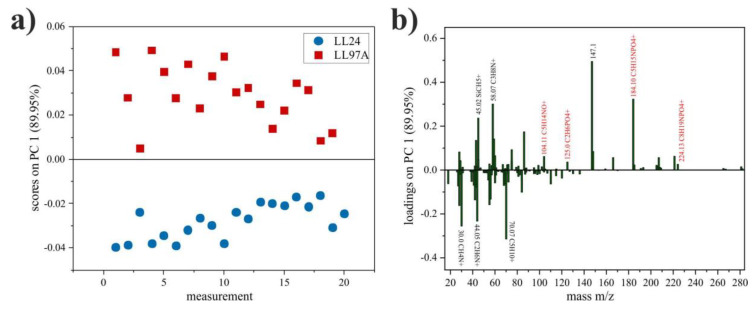
PCA scores plot of LL24 and LL97A cell lines (**a**) and corresponding loadings plot for PC1 with peaks named (black), with the highest differences in intensity and (red) fragments of phosphatidylcholine (**b**).

The representative m/z spectra, recorded for LL24 and LL97A cell (Figure 7) present similar patterns of peaks, with the same m/z positions and only slightly varied intensities. Therefore, to provide a quantitative analysis of even very subtle differences in the chemical composition of examined fibroblasts, a complex PCA analysis was performed (Figure 8).

As seen in Figure 8a, the first principal component (PC1) shows the greatest variation (89.95%) between spectra collected for LL24 (negative scores) and LL97A (positive scores) cell lines. Based on the loadings plot (Figure 8b), it can be seen that the positive scores on PC1 are related with secondary ions, with m/z = 147.1 and m/z = 184.1 (C_5_H_15_NPO_4_^+^) identified as a major cholesterol [39] and phosphatidylcholine [40] fragments, respectively. Moreover, closer inspection of loadings plots also revealed three other fragments (m/z = 104.11, C_5_H_14_NO^+^; m/z = 125.0, C_2_H_6_PO_4_^+^; m/z = 224.13, C_8_H_19_NPO_4_^+^), of phosphatidylcholine, which loaded PC1 positively.

**Figure 9:**
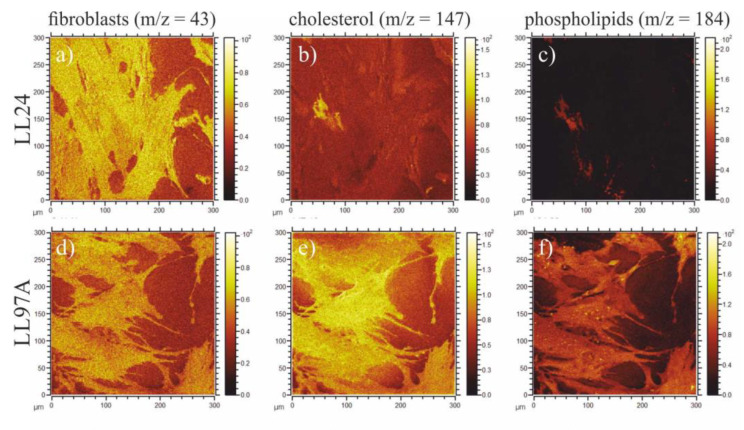
Composition maps corresponding to the total cell area (m/z = 43, **a**,**d**), cholesterol (m/z = 147, **b**,**e**) and phospholipids (m/z = 184, **c**,**f**) recorded for LL24 (upper row) and LL97A (bottom row) cell line.

Finally, the spatial distribution of cholesterol (m/z = 147) and phospholipids (m/z = 184) in cells were traced on composition maps, also provided by ToF-SIMS technique (Figure 9), showing a significant difference in the distribution of both species recorded for healthy (upper row) and IPF-derived (bottom row) fibroblasts.

## 4. Discussion

Our study aimed to verify whether the physicochemical properties of lung fibroblasts, modified by substrate elasticity, can be used to discriminate between normal and fibrotic cells.

### 4.1. Fluorescence

The stiffness of the substrate is known to impact adhesion and proliferation of cells [41,42,43,44,45,46,47]. Our previous research, performed for PDMS elasticity changing stepwise from 600 kPa to 1.5 MPa, showed that substrate stiffness affects the behavior of normal and IPF-derived fibroblasts, influencing the number, shape, and arrangement of cells after 72 h culturing [35]. To elaborate on how fibroblasts behave in a distinct mechanical environment, two PDMS substrates characterized by 600 kPa (referred here as a soft PDMS substrate) and 1.5 MPa (a stiff PDMS) were chosen. The surfaces of these PDMS substrates were not modified with any ECM proteins. The adhesion of fibroblasts to bare PDMS surface proceeds through serum proteins, such as arginine–glycine–aspartic acid sequence (RGD, [48]) from the culture medium, which provides an adhesion matrix for cells, enhancing their capacity to interact and spread [49]. The spreading of fibroblasts was monitored by fluorescence imaging after 24 h, 72 h and 144 h of culture (Figure 1 and Figure 2).

On soft PDMS substrate (600 kPa), normal (LL24, Figure 1), as well as IPF-derived (LL97A, Figure 2), lung fibroblasts grow and proliferate similarly to the fibroblasts culture on a control glass sample. Their growth reaches a state of a confluent monolayer already after 72 h. Cells are flat, with a spindle-like shape, which is typical for fibroblasts.

In turn, on the stiff PDMS substrate (of 1.5 MPa), instead of spreading, cells tend to aggregate and to form agglomerates, with cells growing on each other, as shown in Figure 3. A similar arrangement of fibroblasts was reported for cells cultured in adverse environments, where the cell–cell interactions are favored over cell-substrate ones [50,51,52,53]. Although the aggregation of cells may affect their biological functions [50,51,52,53], it is irrelevant for our goal, i.e., the discrimination between normal and fibrotic lung fibroblasts.

### 4.2. MTS

For healthy lung fibroblasts, the MTS tests show that the number of normal cells is reduced on the stiffer substrate. In contrast, for IPF-derived fibroblasts, the MTS analysis does not reveal any significant difference between the number of IPF-derived cells cultured on stiff and soft PDMS, concluded for fluorescence micrographs, indicating that substrate elasticity affects the arrangement of fibrotic cells rather than their ability to proliferate. The observed different response of both cell types to the substrate stiffness suggests that the internal structure of cells, modified for fibroblasts for ILD disorders, as well as the postulated alteration of their mechanical properties [28,29], influence the cellular behavior and adaptability to the external conditions and might be an important factor in enabling discrimination between healthy and normal cells.

### 4.3. Elasticity

The cytoskeleton organization, as well as the contractility of lung fibroblasts originating from IPF, differs from the normal lung tissue [28]. Knowing that alterations in actin filaments can be related to alterations in the mechanical properties of cells measured by AFM [54,55,56], the Young modulus of lung fibroblasts was determined using AFM-based force spectroscopy [37]

For both cell lines, an increase of Young’s modulus with time is observed, especially for cells cultured on softer PDMS substrate. Here, elasticity modulus doubles for normal fibroblasts and becomes four times larger after 144 h of incubation for IPF-derived ones. This effect may be related to the presence of neighboring cells, which affects Young’s modulus even for AFM measurements carried out on individual cells. The recorded moduli imply that normal fibroblasts remain more rigid as compared to their fibrotic counterparts. Observed difference may indicate the impairments of IPF-derived fibroblasts induced by the fibrosis process, but it can be also linked with the donor age, different for both cell lines. To resolve this issue, extended studies for patient-derived fibroblasts are required.

Observed values of moduli are much higher than the physiological range of the Young modulus of pulmonary tissue (~2–10 kPa) reported elsewhere [24,25,57,58]. Moreover, the observed tendency in cell stiffness is opposite to the one reported before—the healthy fibroblasts are stiffer than the IPF-derived ones. These results are not intuitive, however, IPF-derived fibroblasts differ in important aspects from normal lung tissue-derived fibroblasts, raising the possibility that IPF-derived fibroblasts, like some cancer cell lines, may lose their responsiveness to matrix stiffness [29].

### 4.4. Elasticity Maps

The mechanical properties of cells are substantially different for the cellular membrane and the subcellular components, such as the cytoskeleton or nucleus [59]. The membrane is much softer than other components and mechanical stability is provided by the cytoskeleton, which is also actively involved in the application of forces onto cell-cell and cell-extracellular matrix interactions [60]. Therefore, in addition to the mean elasticity, the spatial distribution of mechanical properties of cells was also analyzed, by recording the elasticity maps of cell fragments (Figure 6).

The observed slight differences in elastic properties between normal and IPF-derived fibroblasts, corresponding to the internal arrangement of the cytoskeleton, may influence the mechanical properties of the examined cells, which may be therefore considered as potential diagnostic markers.

### 4.5. ToF-SIMS

Inflammatory lung diseases are known to affect the biochemical properties of the tissue. Several reports suggest the critical importance of lipoproteins and cholesterol for normal lung physiology [61], however, the detailed underlying mechanisms of this effect remain unclear [61,62]. Most probably, it is related to the perturbations in biochemical homeostasis of the pulmonary surfactant [63], which consists of approximately 90% lipids and 10% proteins, and plays an important role in maintaining normal respiratory mechanics [64]. Moreover, an aberrant deposition of extracellular matrix (ECM) constituents, such as glycosaminoglycans, is characteristic of IPF [65]. Furthermore, diffuse pulmonary ossification (DPO), i.e., calcification in a collagen matrix leading to bone tissue formation, in the lung parenchyma is common in patients with fibrosing ILD, especially with IPF [66,67].

Taking into account the biochemical changes occurring at the cell surface, we test the possibility of discrimination between normal and fibrotic fibroblasts, based on their chemical composition using ToF-SIMS. The representative m/z spectra, recorded for LL24 and LL97A cell (Figure 7), present similar patterns of peaks, with the same m/z positions and only slightly varied intensities. Therefore, to provide a quantitative analysis of even very subtle differences in the chemical composition of examined fibroblasts, a complex PCA analysis was performed.

In turn, ToF-SIMS analysis does not show any difference in the intensities of Ca (m/z = 39.96) and CaO (m/z = 56.96) peaks recorded for both cell lines, indicating a similar amount of Ca for them (Supporting Information, Figure S1), which is in accordance with the glycosaminoglycans and Ca staining (Supporting Information, Figure S2). The surface coverage is comparable for both cell lines and equals to 0.69 ± 0.07 and 0.61 ± 0.05 for healthy and IPF-derived fibroblasts, respectively (cf. Figure 8a,d), therefore, the potential contribution of media components, such as choline, inositol or glutamine to the observed difference is minimized. Moreover, the obtained result is consistent with literature data, indicating that IPF disorder is associated with the disturbance in the biosynthesis and metabolism of cholesterol [62]. Furthermore, the intensity of signals characteristic for phosphatidylcholine [68,69] points to a higher amount of phospholipids for IPF-derived fibroblasts. This effect is intuitive, as oxidized phospholipids are known to influence inflammatory processes [70] and to be highly abundant in the pulmonary surfactant lipid system, disturbed for ILD diseases [62,63,64,69].

Finally, the spatial distribution of cholesterol (m/z = 147) and phospholipids (m/z = 184) in cells was traced on composition maps, also provided by ToF-SIMS technique (Figure 9).

Figure 9a,d show substrate coverage by normal and IPF-derived fibroblasts, respectively. Fibroblasts are visualized here by m/z = 43 signal, present for both cell lines, and not contributing to their differentiation in PCA analysis (Figure 8b). The comparison of maps tracing cholesterol (Figure 9b,e) and phospholipids (Figure 9c,f) shows a significant difference in the distribution of both species recorded for healthy (upper row) and IPF-derived (bottom row) fibroblasts. First of all, their amount is noticeably lower for healthy cells and secondly, their location is changed. For IPF-derived fibroblasts, cholesterol and phospholipids are distributed homogeneously in the whole cell area, whereas for healthy ones, they accumulate on the edge of the cell. The potential contribution of media components, such as choline and inositol, being precursors of phospholipids [71] (m/z = 184) or glutamine to m/z = 147 [72] can be neglected, as the recorded differences in their intensities correspond only to the cell area and remain constant for the substrate not covered with fibroblasts. Based on composition maps recorded using ToF-SIMS and elasticity maps, examined using AFM, no direct relationship between the chemical composition of cells and their mechanical properties may be concluded. The different distribution of cholesterol and phospholipids seems not to be crucial for either the overall mechanical properties of the cell or their spatial distribution. These results, although not intuitive, are in agreement with literature data when the stiffening of cells is observed for cholesterol depletion [73,74,75].

## 5. Conclusions

In our previous work, we have shown, that the stiffness of the substrate, changing from 1.5 MPa to 600 kPa, significantly affects the behavior of both normal and IPF-derived lung fibroblasts, indicating that elastic moduli of cells may play an important role in the development of lung fibroblasts also in the elasticity range far from the physiological one. This motivated us to expand the study and test in details the impact of substrate stiffness on physicochemical properties of normal (LL24) and IPF-derived (LL97A) lung fibroblasts. The growth and viability of cells were recorded using fluorescence microscopy and MTS tests respectively, for culture times ranging from 24 h up to 144 h. For normal fibroblasts, both fluorescence imaging and MTS tests show that the number of normal cells is significantly reduced on the stiffer substrate. In contrast, for fibrotic fibroblasts, MTS analysis does not reveal any remarkable difference between the number of IPF-derived cells cultured on stiff and soft PDMS, whereas fluorescence images show a significant variation of their density. These results indicate that substrate elasticity affects the arrangement of fibrotic cells rather than their ability to proliferate. Additionally, the measurements of elastic modulus of cells, performed using atomic force microscopy working in a force spectroscopy mode showed that normal fibroblasts remain more rigid as compared to their fibrotic counterparts. Observed difference may indicate the impairments of IPF-derived fibroblasts induced by the fibrosis process, but it can be also linked with the donor age, different for both cell lines. To resolve this issue, extended studies for patient-derived fibroblasts are required. Moreover, elasticity maps show that the spatial distribution of mechanical properties differs for both examined cell lines. The chemical properties of normal and IPF-derived lung fibroblasts were inspected using time-of-flight secondary ion mass spectrometry (ToF-SIMS), combined with principal component analysis (PCA), which allows to detect even subtle differences between the samples. As a result, significant differences in the distribution of cholesterol and phospholipids were recorded for healthy and IPF-derived fibroblasts, whereas no differences in Ca abundance were observed.

The observed different response of both cell types to the substrate elasticity suggest that the internal structure of cells, modified for fibroblasts for ILD disorders, as well as the postulated alteration of their mechanical properties influence the cellular behavior and adaptability to the external conditions and might be therefore be seriously considered as an important factor while constructing a diagnostic tool, enabling the fast and reliable identification of IPF fibroblasts. However, before applying such a diagnostic tool for a wide use in medical laboratories, a special protocol for the collection of cells from patients’ tissue must be developed. Although the proposed diagnostic method is not straightforward, we believe that it is worth considering, as nowadays, there is no fast and reliable diagnostic method enabling easy recognition of IPF, which is very often misidentified with other Interstitial lung diseases (ILDs), especially NSIP. Therefore, in our opinion, it seems to be very perspective, as it enables univocal, easy, cheap, and relatively fast diagnosis. Even with the separate step of cell line derivation from the patient’s tissue, the diagnostic process should not exceed a few weeks, which is significantly shorter than the current procedures. Moreover, it is based on the analysis of cell stiffness, which is a measurable, objective parameter, and therefore reduces the possibility of the incorrect interpretation of the results.

## Supplementary Materials

The following are available online at https://www.mdpi.com/1996-1944/13/20/4495/s1, Figure S1: Intensity of Ca (m/z = 39.96) and CaO (m/z = 56.96) signals recorded using ToF-SIMS for LL24 (blue) and LL97 (red) cell line. Error bars indicate the SD (n = 8); Figure S2: Ca staining with alizarin red (left part of image) and glycosaminoglycans staining with alcian blue (right part of the image) recorded on soft (left column) and stiff (central column) PDMS as well as on the control sample (right column) for LL24 (a-c) and LL97A (d-f) cells.
