# Supplementary material for: Effect of Substrate Stiffness on Physicochemical Properties of Normal and Fibrotic Lung Fibroblasts
Source: Materials (Basel). 2020 Oct 10;13(20):4495. doi: 10.3390/ma13204495 (PMC7600549; doi:10.3390/ma13204495)
Supplement: Supplementary file 1 [file materials-13-04495-s001.pdf]

materials

Supplementary Materials

Effect of Substrate Stiffness On Physicochemical Properties of Normal and Fibrotic Lung Fibroblasts

Joanna Raczkowska [1,*], Barbara Orzechowska [2], Sabina Patryas [1], Kamil Awsiuk [1], Andrzej Kubiak [2], Masaya Kinoshita [3], Masami Okamoto [3], Justyna Bobrowska [2], Tomasz Stachura [4], Jerzy Soja [4], Krzysztof Sładek [4] and Małgorzata Lekka [2]

[1] The Marian Smoluchowski Institute of Physics, Jagiellonian University, Łojasiewicza 11, 30-428 Kraków, Poland; sabina.patryas@student.uj.edu.pl (S.P.), kamil.awsiuk@uj.edu.pl (K.A.)
[2] Institute of Nuclear Physics Polish Academy of Sciences, Radzikowskiego 152, 31-342 Kraków, Poland; barbara.orzechowska@ifj.edu.pl (B.O.), andrzej.kubiak@ifj.edu.pl (A.K.), justyma.bobrowska@ifj.edu.pl (J.B.), malgorzata.lekka@ifj.edu.pl (M.L.)
[3] Toyota Technological Institute, 2-12-1 Hisakata, Tempaku, Nagoya, 468-8511, Japan; sd18408@toyota-ti.ac.jp (M.K.), okamoto@toyota-ti.ac.jp (M.O.)
[4] 2nd Department of Internal Medicine, Jagiellonian University Medical College, Jakubowskiego 2, 30-688 Kraków, Poland; tomasz.stachura@interia.pl (T.S.), jerzysoja@op.pl (J.S.), mmsladek@cyf-kr.edu.pl (K.S.)
* Correspondence: joanna.raczkowska@uj.edu.pl

1. Glycosaminoglycans and Ca Staining

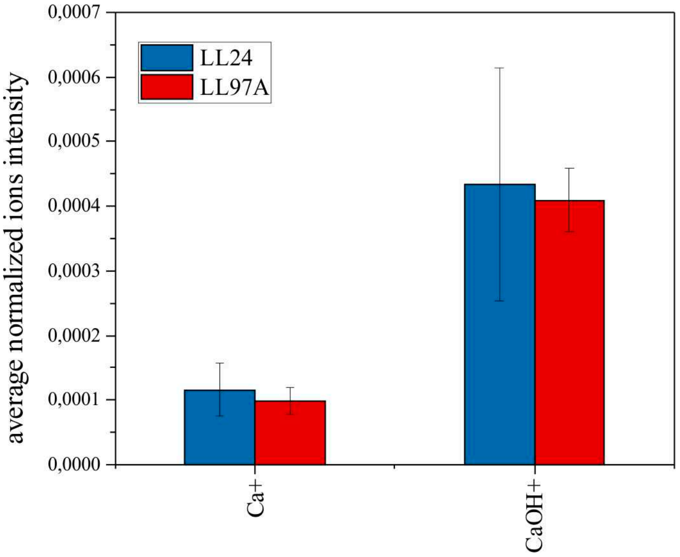

Figure S1. Intensity of Ca (m/z = 39.96) and CaO (m/z = 56.96) signals recorded using ToF-SIMS for LL24 (blue) and LL97 (red) cell line. Error bars indicate the SD (n = 8).

ToF-SIMS analysis for 144h culture time (Figure 11) also do not show any difference in the intensities of Ca (m/z = 39.96) and CaO (m/z = 56.96) peaks recorded for both cell lines, indicating similar amount of Ca for them.

Aberrant deposition of extracellular matrix (ECM) constituents, such as glycosaminoglycans is characteristic for IPF. Also diffuse Pulmonary Ossification DPO, i.e. calcification in a collagen matrix leading to bone tissue formation, in the lung parenchyma is common in patients with fibrosing ILD, especially with IPF. To test possibility to use glycosaminoglycans or Ca as IPF biomarkers for an application in a diagnostic tool, the distribution of both species in healthy and IPF-derived fibroblast was traced using optical microscopy for cells stained with alizarin red and alcian blue, which are the standard dies enabling Ca and glycosaminoglycans visualization.

Images recorded after 72h of culturing on PDMS substrates with modified elastic properties as well as on glass (Figure S2) do not show any influential differences, neither in Ca amount nor

distribution between healthy and IPF-derived fibroblasts. Similar results are observed for glycosaminoglycans. Observed lack of difference in Ca distribution between cells could be potentially caused by the culturing time, too short for effective calcification process.

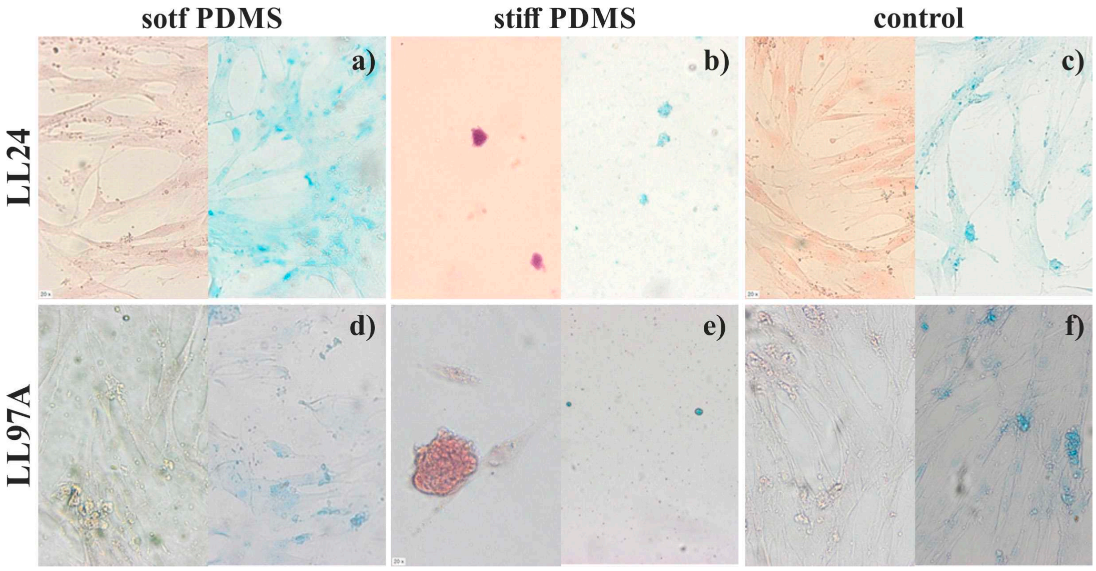

Figure S2. Ca staining with alizarin red (left part of image) and glycosaminoglycans staing with alcian blue (right part of the image) recorded on soft (left column) and stiff (central column) PDMS as well as on the control sample (right column) for LL24 (a-c) and LL97A (d-f) cells.

2. Calcium and Glycosaminoglycans Staining—Methodology

After 48 h, 72 h and 144 h of culture, cells were rinsed two times with PBS (Sigma), fixated with 3.7% paraformaldehyde (Sigma) for 20 min and rinsed 3 times with PBS.

Directly before $Ca2^{+}$ staining cells were additionally rinsed two times with distilled water. Staining with 40 mM Alizarin Red Staining Solution (Sigma-Aldrich) were performed for 5 min. Afterwards cells were rinsed three times with distilled water and calcium deposits were imaged with Olympus IX53 inverted microscope. To visualize glycosaminoglycans, which are characteristic for cartilage tissue, cells were rinsed two times with distilled water and then stained with 1% w/v Alcian Blue Staining Solution (Sigma-Aldrich) for 30 min. Afterwards cells were rinsed three times in distilled water and then imaged with Olympus IX53 inverted microscope. To prove the reproducibility of the results the experiments were repeated at least three times for each cell line and a time-point. For each experimental sequence two or three identical samples were prepared and measured.

© 2020 by the authors. Licensee MDPI, Basel, Switzerland. This article is an open access article distributed under the terms and conditions of the Creative Commons Attribution (CC BY) license (http://creativecommons.org/licenses/by/4.0/).
